# A bio-functional cryogel with antioxidant activity for potential application in bone tissue repairing

**DOI:** 10.1016/j.heliyon.2024.e37055

**Published:** 2024-08-28

**Authors:** Feng Chen, Wenbiao Zheng, Zeyu Yang, Wei Wang, Jiehe Huang

**Affiliations:** a Department of Orthopedics, Taizhou Municipal Hospital, Taizhou, 318000, Zhejiang Province, PR China; b Department of Tumor Intervention, Taizhou Municipal Hospital, Taizhou, 318000, Zhejiang Province, PR China

**Keywords:** Aerogel, Oligomeric proantho cyanidins, Antioxidant activity, Bio-functional, Tissue engineering

## Abstract

Antioxidant and free radical resistance has been a key concern of tissue engineering. In this study, Hydroxyapatite (HAp) with osteogenic activity and Oligomeric Proantho Cyanidins (OPC) with antioxidant activity were chemically grafted to prepare gelatin-based biofunctional aerogel (GHPOS). SEM results confirmed that these aerogels exhibited obvious macroporous structure and could provide a suitable microenvironment for bone cell growth. The addition of HAP-PEI-OPC made it have good antioxidant activity, and the cell results proved that the aerogel prepared in this study had good cytocompatibility and did not produce cytotoxicity. The addition of nanoparticles played an important role in the activity of 3T3-E1. The results showed that these bioactive aerogel scaffolds have potential applications in bone tissue engineering.

## 1 Introduction

Gelatin (Gel) is a hydrolyzed product of collagen, which retains the chemical structure of collagen and is a polymer composed of a variety of amino acids [[1], [2], [3], [4]]. It has good water absorption, water retention and degradation properties, and good adhesion properties, and has a wide range of applications in the biomedical field [5]. Its biggest advantages are a wide range of sources, low price, good biocompatibility, can be completely degraded and absorbed in the body, non-toxic to the human body without antigen, so gelatin is an ideal tissue engineering material. In recent years, with the development of tissue engineering, gelatin has attracted much attention because of its unique biological properties [[6], [7], [8]].

Oligomeric Proantho Cyanidins (OPC) is a kind of flavonoids extracted from plants [9,10]. OPC have been widely proven to have antioxidant, anti-myocardial ischemia-reperfusion injury, anti-atherosclerosis, protection of vascular endothelial cells and other pharmacological activities. A series of studies have been conducted on the related antioxidant activity of procyanidins. For example, Hongliang Jiang et al. have compared the effect of procyanidine (PA) on the properties of the electrospun gelatin membranes [11]. The results proved that the PA-crosslinked membranes displayed the highest resistance to pepsin degradation, and fibroblast cells could migrate deeper into the interior of the membranes due to the good preservation of the fibrous structure during the cell culture process. Yu-Ming Kang et al. have constructed a rat model of renal vascular hypertension, the results shown that PVN infusion of OPC inhibited the progression of renovascular hypertension through its potent anti-oxidative and anti-inflammatory function in the PVN [10]. However, when the newly developed biomaterials are used for tissue repair, they should have properties and be biocompatible [12]. More importantly, When it comes to the formation of biological structures, one of the most significant challenges is to ensure that specific physicochemical and biological signals exist in a harmonious manner to regulate the activities of cells [13].

Hydroxyapatite (HAp) is the main inorganic component of human and animal bones [[14], [15], [16], [17]]. It can achieve chemical bonding with body tissue on the interface, release harmless ions to the body, participate in metabolism, promote the repair of defect tissue. However, although HAp is similar to human bone and has good osteogenic effect, its biological activity is limited [18,19]. At present, HAp is mostly used in combination with other active factors to achieve better tissue repair effect [14,17].

As we all know, gelatin is already used in biomedical applications due to its biocompatibility and biodegradability, but enhancing it with bioactive nanoparticles tailored for specific therapeutic functions (osteogenic and antioxidant) enhances its utility in bone tissue engineering. In this work, the chemical grafting of HAp, which is known for its osteogenic properties, with OPC, which are recognized for their antioxidant activities, into nanoparticles is a novel approach. Specifically, OPC was introduced into HAp to obtain nanoparticles with antioxidant activity, and then the active particles were loaded into gelatin aerogel by freeze-drying technology to obtain Gel/HAp-PEI-OPC scaffold (GHPOS) with biological activity. The microstructure, mechanical properties and swelling properties of aerogel were characterized. The free radical scavenging ability of different aerogels were evaluated by DPPH scavenging effect. In addition, the bioactivity of the aerogel to cells was tested by cell assay, and the biocompatibility of the aerogel was evaluated. These results indicated that aerogels have good antioxidant activity and good cell activity, and have potential application in bone tissue engineering.

## 2 Materials and methods

### 2.1 Materials

Gelatin (Gel, type B from porcine skin), hydroxyapatite (HAp, with 99 % purity), polyetherimide (PEI), oligomeric proantho cyanidins (OPC, with 95 % purity), 2,2-diphenyl-1-(2,4,6-trinitrophenyl)-Hydrazine group (DPPH, with 97 % purity) are purchased from Sigma-Aldrich Trading Co., Ltd. (Shanghai, China). For cell culture, Dulbecco's modified eagle medium (DMEM), fetal bovine serum (FBS), pancreatic enzyme and other relevant reagent are obtained from Hyclone Trading Co., Ltd. (Shanghai, China). Calcein-AM and propidium iodide (PI) are utilized to identify live/dead cells.

All the above-mentioned solvents and reagents are of analytical grade and can be used without further purification.

### 2.2 Preparation of biofunctional aerogel

#### 2.2.1 Preparation of biofunctional nanoparticle

100 mg HAp was ultrasonically dispersed in 50 mL DI water forming a homogeneous presolution. After that, 200 mg PEI was added into the HAp solution. The mixed solution was stirred for 6 h and sonicated for 30 min to form HAp-PEI modified mixture solution. Then 50 mg OPC was added in the mixed solution and the solution was continuously stirred using a stirrer for 3 days until completely reacted. Finally, HAp-PEI-OPC nanoparticles were obtained via centrifugation (8000 r/min) and freeze drying.

#### 2.2.2 Preparation of aerogel

1 g gelatin was dissolved in 10 mL deionized water at 50 °C to obtain gelation solution (w/v = 10 %). After stirring until clarified, HAp-PEI-OPC nanoparticles were added into gelation solution and stirred for 8 h to form Gel/HAp-PEI-OPC solution. Through freeze drying, the target aerogels were obtained and named as Gel/HAp- PEI-OPC scaffold (GHPOS). Additionally, a pure Gel scaffold (GS) and an HAp-loaded Gel scaffold (GHS) were named for aerogel, respectively. All processes were managed at around 25 °C and 25 % ± 5 % relative humidity.

### 2.3 Characterization of aerogel

The micro morphology of the prepared aerogel (GS, GHS and GHPOS) are observed via scanning electron microscope (SEM, Phenom XL, Netherlands) operating with sputter gold plating for 35 s at 5 mA at an accelerating voltage of 10 kV.

The ethanol displacement method was applied for confirming the porosity of scaffolds. The samples were dipped in absolute ethanol, and the bubble was removed. The porosity was determined by,

$$\text{porosity}\;\left(\%\right)=\frac{V_{1}-V_{3}}{V_{2}-V_{3}}\times 100\%$$

where *V*_*1*_ expresses the volume of known ethanol; *V*_*2*_ stands for the bulk volume of the ethanol impregnated scaffolds and ethanol; *V*_*3*_ is the volume after removed the scaffolds.

For mechanical testing, the aerogel was prepared into a cylinder of 1.5 cm × 1.5 cm, and the compression cycle test for the aerogel under 60 % strain was measured with a tensile machine (HY-025CS, Shanghai Hengyu Instrument Co., Ltd, China). The samples were compressed for 10 times at a compression speed of 1 mm/min. Each experiment was repeated three times.

The swelling performance of aerogel was tested by the swelling rate of buffer solution in different time. Briefly, GS, GHS and GHPOS were immersed into PBS and DMEM at 37 °C with speed of 100 rpm, respectively. At specific time point, the samples were taken out at 12 h and 24 h from PBS and DMEM, respectively. The weight of scaffolds with different time were measured to observe the swelling state of aerogel [[20], [21], [22]].

### 2.4 Oxidation resistance of aerogel

The antioxidant efficiency of aerogel was evaluated by scavenging 1,1-diphenyl-2-picrylhydrazine (DPPH) free radicals [23,24]. 100 μM DPPH solution was diluted with anhydrous ethanol. 1 mL of the sample solution was added to 3 mL DPPH solution for stirring, and placed in the dark for half an hour. The absorbance of DPPH was determined by UV–vis spectrophotometry (517 nm).

$$\text{DPPH}\;\text{clearance}\;\text{rate}\%=\frac{D-D_{H}}{D}\times 100\%$$

Where D and D_H_ are the absorbance of the blank sample and the absorbance of the target aerogel, respectively.

### 2.5 Cell experiment

3T3-E1 cells were obtained from Shanghai Cell Bank of Chinese Academy of Sciences (Shanghai, China) and cultured with growth medium consists of dulbecco's modified eagle medium (DMEM), 10 % fetal bovine serum and 1 % penicillin/streptomycin. 3T3-E1 cells were utilized to measure the bioactivity of GS, GHS and GHPOS aerogels [25,26]. Before seeding, the samples were steam sterilized with 75 % alcohol and then irradiated with ultraviolet light for 24 h at room temperature. This dual approach not only ensures thorough sterilization but also prevents sample denaturation, which can occur due to prolonged exposure to either method. Utilizing 75 % ethanol, rather than pure ethanol, mitigates the issue of rapid evaporation, maintaining a consistent ethanol vapor environment around the sample. Therefore, 75 % ethanol was chosen for this procedure. Then, the sterilized aerogel was placed in a 24-well plate and added with 1 ml medium for incubation at 37 °C. After incubation, the samples were removed and the surrounding medium was collected as sample solution. After that, cells were mixed with medium, and added to each well at a density of 1.5 × 10^4^ cells/well, each sample was repeated three times. The culture medium was maintained to be replaced every 2 days. Cover slip was used as controls.

The cell viability of 3T3-E1 cells were measured via cell counting kit-8 (CCK-8). 3T3-E1 cells were cultured in GS, GHS and GHPOS for 1, 3, and 5 days, respectively. At the set time, the samples were washed with PBS and CCK-8 solution were added to the culture plate. Then the well plate was placed in CO_2_ incubator and cultured for 2 h, The OD value was measured with a microplate reader at a wavelength of 450 nm. Additionally, on the third day, the cell viability of the samples was determined with a live and death staining kit and observed by fluorescence microscope [27].

### 2.6 Protein absorption evaluation of aerogel

The sterilized aerogel was co-cultured with 3T3-E1 cells for 12 h. At the set time, the cultured aerogels were removed from the cell culture medium and washed with PBS for three times. Then, the aerogels were incubated with 1 mL FBS at 37 °C for 72 h. The protein detection system (GloMax Multi + In vitro protein Expression System) was used to measure the concentration of FBS before and after adsorption at 280 nm [[28], [29], [30]].

### 2.7 Statistical analysis

One way analysis of variance (ANOVA) was used to evaluate the data in this research. The experimental data is analyzed using Origin 2018 pro, and the results are expressed as the mean ± standard deviation. p < 0.05 was considered statistical significance, while * indicates p < 0.05, ** indicates p < 0.01, *** indicates p < 0.001.

## 3 Results and discussion

The difficulty of bone tissue defect repair lies in the recruitment of bone cells and the maintenance of the vitality of original bone cells [31,32]. It has been reported that the original osteocytes of damaged bone tissue are prone to apoptosis during growth process, resulting in failure of bone repair. This is mainly due to the low antioxidant activity of damaged bone tissue, resulting in disorder of cell microenvironment and ultimately cell death. Therefore, antioxidant activity is an important factor in bone tissue repair.

**Fig. 1:**
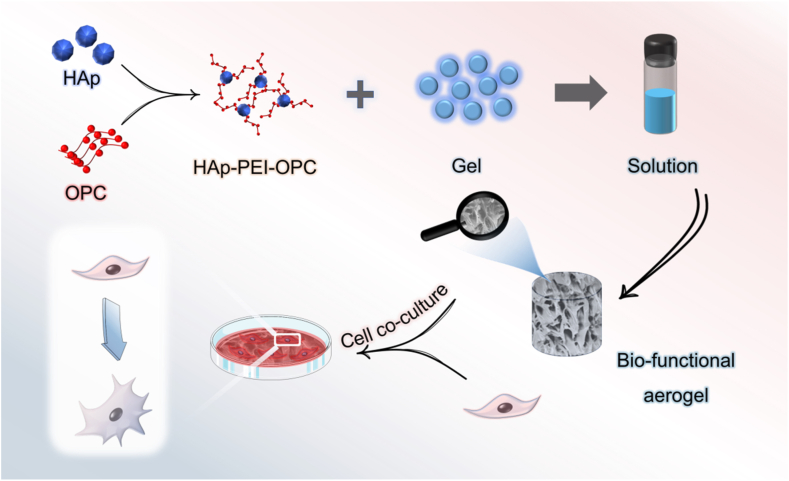
Schematic illustration of the preparation for bio-functional aerogel.

Based on this, a three dimensional porous aerogels with antioxidant activity has been designed in this research (Fig. 1). OPC was loaded on the surface of HAp to form a bioactive nanoparticle with antioxidant activity and mixed with Gel. This aerogels could be used for extracellular matrix adhesion, proliferation, in vitro or in vivo growth and differentiation of host cells, and provide an ideal microenvironment for intercellular communication.

### 3.1 The characterization of aerogel

**Fig. 2:**
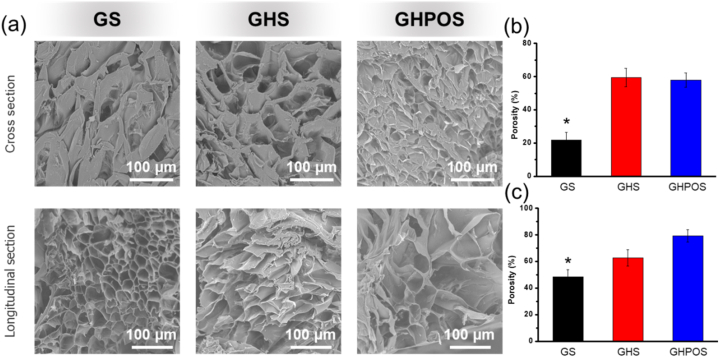
(a) Characterization of the microscopic morphology of GS, GHS and GHPOS; (b) Porosity of cross section for GS, GHS and GHPOS; (c) Porosity of longitudinal section for GS, GHS and GHPOS.

In tissue engineering, researchers have confirmed that pore microstructure plays an important role in cell adhesion and proliferation [33]. Proper pore structure can promote cell growth into scaffold and improve cell vitality. In this research, biofunctional aerogels were prepared via freeze-drying method. Scanning electron microscope (SEM) was used to observe the micro morphology of GS, GHS and GHPOS (Fig. 2a). The results showed that the cross section of the aerogel was porous and there were interconnected macro porous structures in the scaffold. Furthermore, the SEM of cross section exhibited that the addition of HAp and HAP-PEI-OPC nanoparticles had a significant effect on the pore structure of gelatin scaffold. The addition of particles increased the pore density and porosity of GHS and GHPOS while compared with GS (Fig. 2b). While compared with GS, the incorporation of HAp significantly increased the pore size of GHS. This can be explained by the fact that the addition of nanoparticles changed the crystal lattice of gelatin, resulting in more obvious pore structure in the freeze-drying process.

Additionally, longitudinal section SEM of GS, GHS and GHPOS showed a significantly different morphology. Compared with cross section, longitudinal section performed the higher pore density and lower pore size (Fig. 2c). The pore structure of these aerogels showed a more regular and arranged character. Similarly, with the addition of nanoparticles, the longitudinal morphology also showed a larger pore structure. This pore structure facilitates the transport of oxygen and nutrients and the growth of cells, which may have a positive effect on cell growth.

### 3.2 Mechanical properties of aerogels

Bone tissue is constantly subjected to diverse mechanical forces in physiological conditions, including gravitational loads, muscle contractions, and pressure from surrounding tissues. Consequently, scaffold materials employed for bone repair must possess sufficient mechanical strength to withstand these forces. This resilience is crucial to prevent scaffold rupture or displacement, which could lead to reinjury or delayed healing. Thus, the mechanical properties of bone scaffolds play a critical role in ensuring successful bone regeneration.

**Fig. 3:**
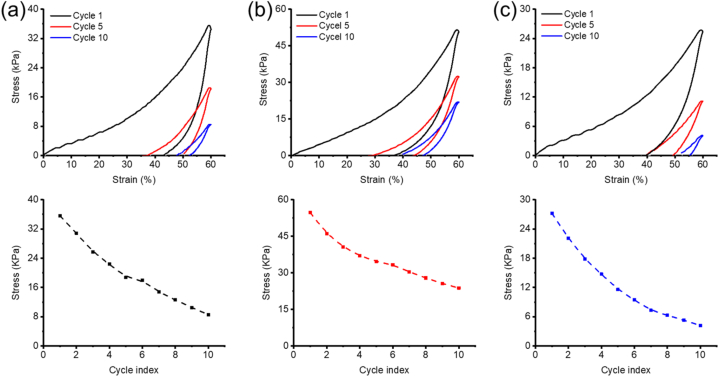
Mechanical test of aerogel. Cyclic loading-unloading stress-strain curve of aerogel at 60 % strain of GS (a), GHS (b) and GHPOS (c) and its stress loss at the 10th cycle.

The mechanical properties of GS, GHS and GHPOS were tested by cyclic loading and unloading tests. Fig. 3 showed the mechanical curve of GS (Fig. 3a), GHS (Fig. 3b) and GHPOS (Fig. 3c) after 10 compression cycles under stress. After the first loading-unloading cycle, the three groups of aerogel showed a large hysteresis loop, indicating that aerogel had a certain degree of loss. This is mainly because aerogel is a large pore structure, which is compressed and collapsed in the compression process [34]. This phenomenon is consistent with SEM image results (Fig. 2). In the fifth and tenth loading and unloading cycles, although the sample still has some losses, the difference between the fifth hysteresis loop and the tenth hysteresis loop is relatively small. It indicated that aerogel had lost stress to a certain extent after the tenth cycle of loading and unloading during the compression process, but still maintained good elasticity.

It was clearly that GHPOS showed the lowest stress during the loading-unloading cycle testing. Compared with GS and GHS, GHPOS exhibited the highest stress loss during the compression cycle, indicating its higher hardness. The addition of HAp-PEI-OPC nanoparticle may increase the hardness of GHPOS. In addition, the active groups on OPC could cross-link with the gelatin, further improving the internal network of the aerogel and thus enhancing its hardness. The increased hardness of the scaffold suggests enhanced resistance to external pressure during bone transplantation, better preservation of its structural integrity, and reduced susceptibility to collapse. This has a beneficial effect on maintaining the integrity of bone tissue sites.

### 3.3 Swelling properties of aerogel

**Fig. 4:**
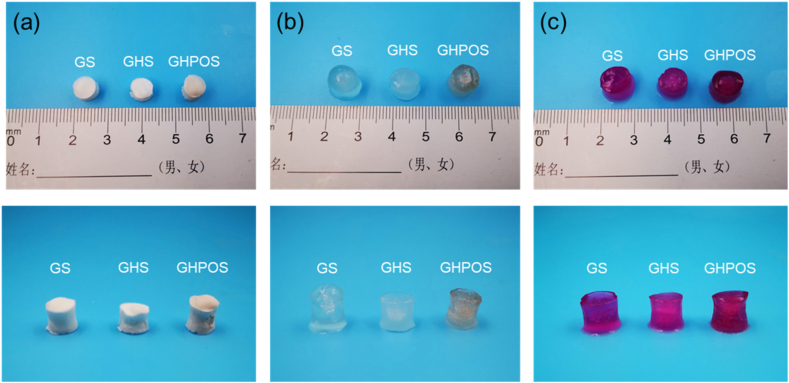
The morphology of the swelling of aerogel in dry (a), PBS (b) and DMEM (c) after 24 h.

Usually, there is a small amount of tissue fluid near the injured tissue, which has a negative effect on tissue repair [35]. In order to provide a good healing environment for injured tissues, aerogel should have good swelling capacity [22]. In this research, two commonly used buffers (PBS and DMEM) were utilized to evaluate the swelling performance of aerogel. Fig. 4a–c exhibited the macromorphology of aerogel before and after swelling for 24 h. The results performed that the volume of aerogels increased to varying degrees after swelling, and the aerogels turned into jelly-like gel.

**Fig. 5:**
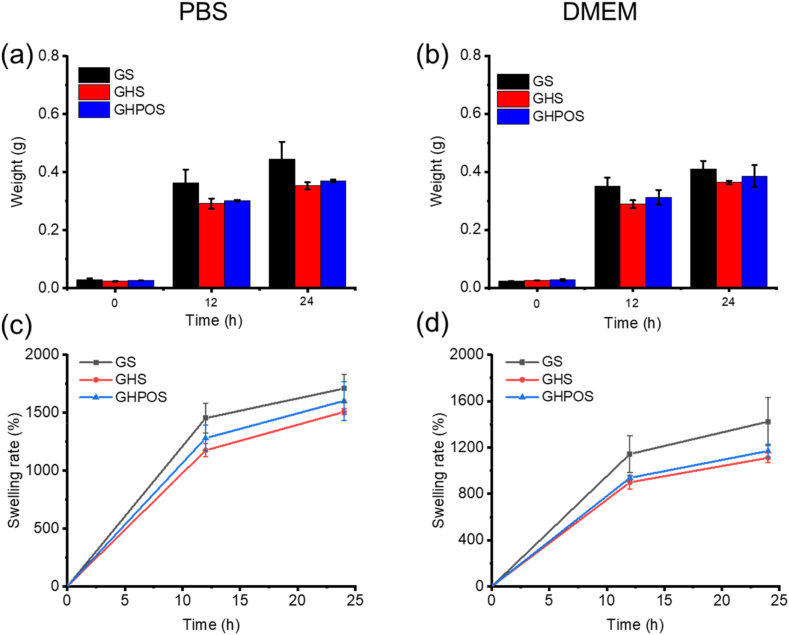
(a–b) The weight of aerogel in different solution (a: PBS, b: DMEM) after 12 h and 24 h; (c–d) Absorption rate of aerogel in different solution (c: PBS, d: DMEM).

Fig. 5 showed the weight change and quantitative analysis of GS, GHS and GHPOS at different swelling time. The weight change of aerogel in PBS and DMEM were shown in Fig. 5a, c and 5b, 5d. The results displayed that the weight of aerogel increased significantly after soaking in PBS and DMEM buffers for 12 and 24 h, and the weight of GS was the highest during the same swelling time while comparing with GHS and GHPOS. This can be explained by the addition of nanoparticle. The swelling rate of GHPOS was more than 1000 % both in PBS and DMEM, and the high swelling rate of aerogel was due to its interconnected pore structure. Although the addition of active nanoparticles reduced the swelling rate, it still had a good macroporous structure, which could absorb the wound exudate and effectively promote tissue repair. The results demonstrated that the scaffold could swell up to 1000 % without rupturing while maintaining excellent structural stability. This confirms that post-implantation, the scaffold can effectively absorb tissue fluid, adhere to the bone tissue, and retain its three-dimensional structure without collapsing or shifting.

### 3.4 Antioxidant properties of aerogel

**Fig. 6:**
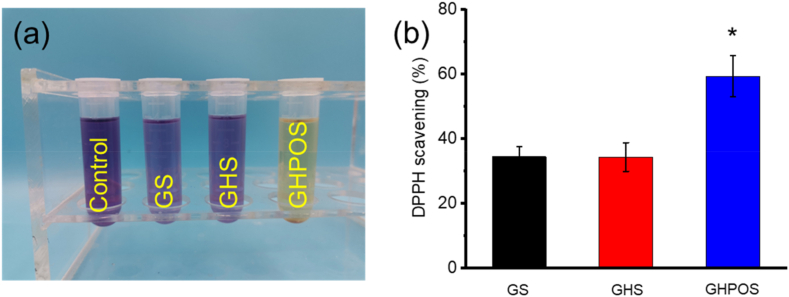
The antioxidant activity of GS, GHS and GHPOS. (a) The antioxidant photograph; (b) DPPH clearance quantitative analysis.

Antioxidant activity is a key factor in promoting micro angiogenesis during bone tissue repair [23,36]. The antioxidant activity can be measured by DPPH removal assay. The results indicated that compared with the control group, GS, GHS and GHPOS had certain antioxidant activity (Fig. 6). The GHS and GHPOS groups became pale purple (Fig. 6a). The loading of OPC significantly improved the free oxygen scavenging ability of aerogel, and the free radical scavenging ability reached more than 60 % within 30 min (Fig. 6b). The enhanced antioxidant activity also further proved the successful graft and introduction of HAp-PEI-OPC particles.

### 3.5 Cell viability of aerogel

**Fig. 7:**
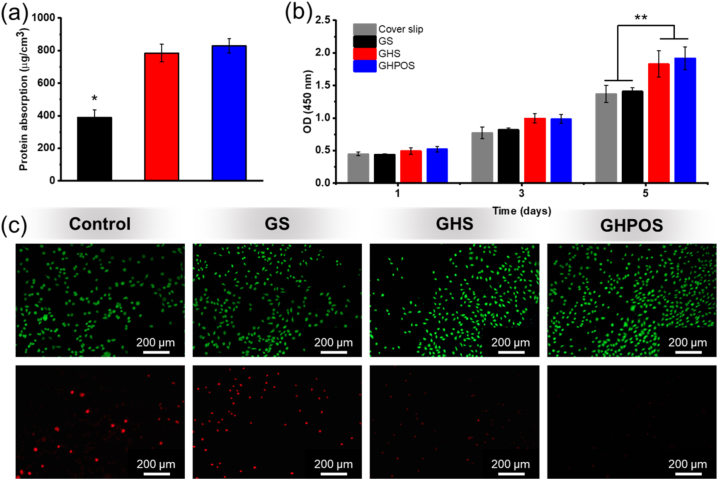
(a) Protein absorption results of GS, GHS, and GHPOS for 72 h, respectively; (b) Viability of 3T3-E1 after culturing for 1, 3, and 5 days with GS, GHS, and GHPOS, respectively; (c) Fluorescence micrographs of 3T3-E1 on GS, GHS, and GHPOS after culturing for 3 days.

Protein adsorption capacity plays an important role in the interaction between cells and matrix materials. Proteins adsorbed on the surface of biomaterials can recognize cells and promote cell behavior and activity. Therefore, the protein adsorption performance of aerogel (GS, GHS, and GHPOS) were evaluated in vitro. The adsorption values of GS, GHS and GHPOS were shown in Fig. 7a. The result informed that HAp-PEI-OPC aerogel exhibited excellent protein adsorption capacity while compared with GS. This may be because the addition of HAp-PEI-OPC increases the porosity of GHPOS and enhances the permeability of protein solution to aerogel.

CCK-8 assay was utilized to test the cytocompatibility of GS, GHS and GHPOS (Fig. 7b). there was no significant difference in cell viability between samples on the day 1. With the increase of culture time, OD values of both 4 groups were improved gradually. After culture for 5 days, the cell viability of GHS and GHPOS were higher than that of control and GS, indicating that the addition of nanoparticles had positive effect on cell viability, especially for bone cells.

The cell viability of 3T3-E1 on different samples can be visually displayed by the staining of live and dead cells. The cytocompatibility of cells on samples can be judged by the number of green cells and red cells in the field of vision. It can be seen from Fig. 7c that 3T3-E1 showed excellent growth activity on both GHS and GHPOS. By comparing GHS and GHPOS, red dead cells could be observed in blank and GS, while almost no dead cells could be observed in GHPOS. Besides, 3T3-E1 showed a lower densely on the blank group than other samples. Gelation has been demonstrated to yield high cell viability and is widely utilized in tissue engineering [8,37]. The addition of HAp and HAp-PEI-OPC can also promote the growth of osteoblast [13,15]. Furthermore, the cells grown on GHPOS showed stronger green fluorescence than those grown on GS and blank group, indicating that the growth activity of cells on GHPOS scaffold was better than that on GS scaffold, and the scaffolds added with modified HAp-PEI-OPC nanoparticles were more conducive to the growth of osteoblasts.

## 4 Conclusions

In response to the antioxidant and osteogenic requirements for bone tissue repair, this study successfully prepared organic-inorganic composite biofunctional GHPOS aerogels with antioxidant effects. The incorporation of HAp-PEI-OPC imparts excellent antioxidant activity to the scaffold, while also endowing it with favorable swelling and absorption properties for buffer solutions. Additionally, the inclusion of nanoparticles enhances the hardness and pore size of the aerogel, thereby promoting bone cell growth into the scaffold. The introduction of inorganic nanoparticles significantly influences the activity of 3T3-E1 cells. These findings affirm the potential application of these bioactive aerogel scaffolds in bone tissue engineering.

## Data availability

The data this work utilized were all available and accessible in the before-noted websites and databases.

## CRediT authorship contribution statement

**Feng Chen:** Writing – original draft, Formal analysis. **Wenbiao Zheng:** Investigation. **Zeyu Yang:** Formal analysis. **Wei Wang:** Software, Funding acquisition. **Jiehe Huang:** Writing – review & editing, Funding acquisition.

## Declaration of competing interest

The authors declare the following financial interests/personal relationships which may be considered as potential competing interests:

Wei Wang reports financial support was provided by the second batch of social development science and technology plan projects in Taizhou City. If there are other authors, they declare that they have no known competing financial interests or personal relationships that could have appeared to influence the work reported in this paper.
